# Digital twin based automated surveillance system for infection cluster detection, outbreak mapping and contact tracing in a tertiary ho

**DOI:** 10.1017/ash.2025.240

**Published:** 2025-09-24

**Authors:** Indumathi Venkatachalam, Xiang Ying Jean Sim, Edwin Philip Conceicao, Weien Chow, Chi Ting Low, Shalvi Arora, Revathi Sridhar, Laura Chan, Nicholas Graves, Yiying Cai, Sean Wu, Isaac Low

**Affiliations:** 1 Singapore General Hospital; 2 Singhealth; 3 Changi General Hospital; 4 National University Hospital Singapore

## Abstract

**Background:** Traditional infectious disease surveillance data have significant lag time limiting their usefulness in infection cluster detection in healthcare settings. Digital twin spatial representation, electronic healthcare data integration and surveillance automation allow for timely cluster detection and facilitate faster outbreak mapping and contact tracing, better informing infection prevention practice. **Method:** 4-Dimensional Disease Outbreak Surveillance System (4D-DOSS) is an automated infectious disease surveillance system developed in Singapore General Hospital (SGH), a 2000-bed tertiary healthcare institution. Electronic patient data (bed allocation and laboratory test results) are integrated onto a digital twin of SGH, and surveillance algorithms are applied for routine surveillance and contact tracing. 4D-DOSS was operationalized in SGH and National Heart Centre Singapore (NHCS) on August 1st, 2024. Active surveillance for carbapenemase producing enterobacterales (CPE) in SGH and NHCS includes contacts of inpatients with CPE carriage. Contact tracing for CPE is done on 4D-DOSS. Primary and secondary contact tracing are algorithmically automated. Spatial and temporal patterns are analyzed to understand transmission networks in outbreaks. Automated email alerts can be sent to clinicians to notify significant test results. **Results:** Contact tracing typically takes two hours per index patient using traditional methods. Contact tracing for CPE using 4D-DOSS takes five minutes per index patient, and multiple index patients can be traced per trace. Based on about 50 COVID-19, CPE and VZV combined exposure events per week in 2023, at 1.92 hours saved per exposure event, there would be a saving of 648 FTE per year, Between August 1st, 2024 and December 31st, 2024, there were eight VRE, eight CPE and 17 acute respiratory viral infection (RVI) clusters in inpatient wards. Selected clusters were viewed during weekly epidemiology rounds to get a better understanding of the transmission network. Outbreak mapping of infection clusters using traditional methods can take up to two days whereas each cluster can be analyzed in 4D-DOSS in under one hour. If four outbreaks are mapped per year, at 47 hours saved per outbreak mapped, the estimated FTE saved is 24 per year.

4D-DOSS has been configured for email alerts for acute RVI in patients in a selected ward since the last week of December 2024. Seven alerts were received in the first week of implementation. **Conclusion:** The comprehensive digital twin-enabled infectious disease surveillance platform enabled an efficient contact tracing and outbreak mapping system and automated surveillance alerts facilitating timely infection prevention measures. This can potentially improve patient outcomes.

